# A Case of Acquired Cerebral Achromatopsia Secondary to Posterior Cerebral Artery Stroke

**DOI:** 10.7759/cureus.14798

**Published:** 2021-05-02

**Authors:** Mohammad Selim, Erica Cichowski, Rachel Kubowicz, Faysal Alghoula, Rammohan Sankaraneni

**Affiliations:** 1 Internal Medicine, Creighton University School of Medicine, Omaha, USA; 2 Neurology, Creighton University School of Medicine, Omaha, USA

**Keywords:** stroke, pca infarction, color blindness, achromatopsia, cerebral achromatopsia

## Abstract

Impairment of color vision is known as “Achromatopsia.” This condition is multifactorial with a myriad of causes, from local at the retinal level to central at the occipital cortex level. The most common causes are inherited conditions. However, acquired achromatopsia has been acknowledged in numerous case reports and studies. Achromatopsia secondary to posterior cerebral artery (PCA) stroke is an extremely rare phenomenon and had been reported in a few case reports. In this case, we report a patient presenting with achromatopsia as the only complaint due to an infarction of the left occipital cortex.

## Introduction

Achromatopsia is the impairment of color vision. This condition is a well-known inherited disorder; however, acquired defects due to multiple local and central causes have been reported [1]. Acquired cerebral disturbances of color perception are rare but long acknowledged, with the first case being described more than a century ago in 1888 by a Swiss ophthalmologist, Louis Verrey [2]. Recent authors have acknowledged its occurrence, but the responsible lesion's precise site has not been thoroughly analyzed. 

Strokes can involve any part of the brain, depending on the geographic distribution of the occluded vessel's cortical territory. The resulting neurological deficit then depends on the functional area impacted by the cerebral event. Posterior cerebral artery (PCA) strokes represent around 10% of all acute ischemic strokes. PCA strokes are most frequently due to lacunar infarction but may be secondary to atherothrombotic and/or cardioembolic etiologies [1]. We present a rare case of acquired color blindness, “achromatopsia,” that resulted from a stroke in the visual cortex of the occipital lobe. The patient did not have any of the other manifestations of a PCA stroke, and her National Institutes of Health Stroke Scale (NIHSS) score was zero. She was out of the window for thrombolytic consideration. Magnetic resonance imaging (MRI) of the brain confirmed the diagnosis, and aspirin and statin therapies were initiated. Color blindness is a rare presentation of PCA stroke, which can be unrecognized, resulting in a delayed diagnosis and treatment.

## Case presentation

A 71-year-old right-handed female with a past medical history of scleroderma, pulmonary fibrosis, and right middle cerebral artery (MCA) territory infarct seven years before this presentation. She was treated with low-dose aspirin and high-dose statin medications, which she stopped 6 months before this presentation. She had fully recovered from the prior stroke without any clinically evident sequelae. There is no history of tobacco or illicit drug use, and she quit drinking alcohol more than six years before this presentation.

The symptoms started the night before the admission when she was walking out of a casino and had difficulty recognizing her vehicle and finding her way back home, which she attributed to exhaustion. The patient noticed her color blindness upon waking up when she looked out of the window and could not see the trees green color. She could see black and white only and could not distinguish between different grades of the same color. The patient denied focal motor weakness, dysarthria, aphasia, gait disturbance, blurry vision, dizziness, headache, or sensory loss. She denied fever, recent trauma, and sick contacts. Upon the presentation, her vital signs were within the normal range. A general examination was unremarkable, and neurological examination showed no focal motor weakness, no sensory abnormalities, no coordination deficits, no cranial nerve abnormalities, and no visual field defects.

**Figure 1:**
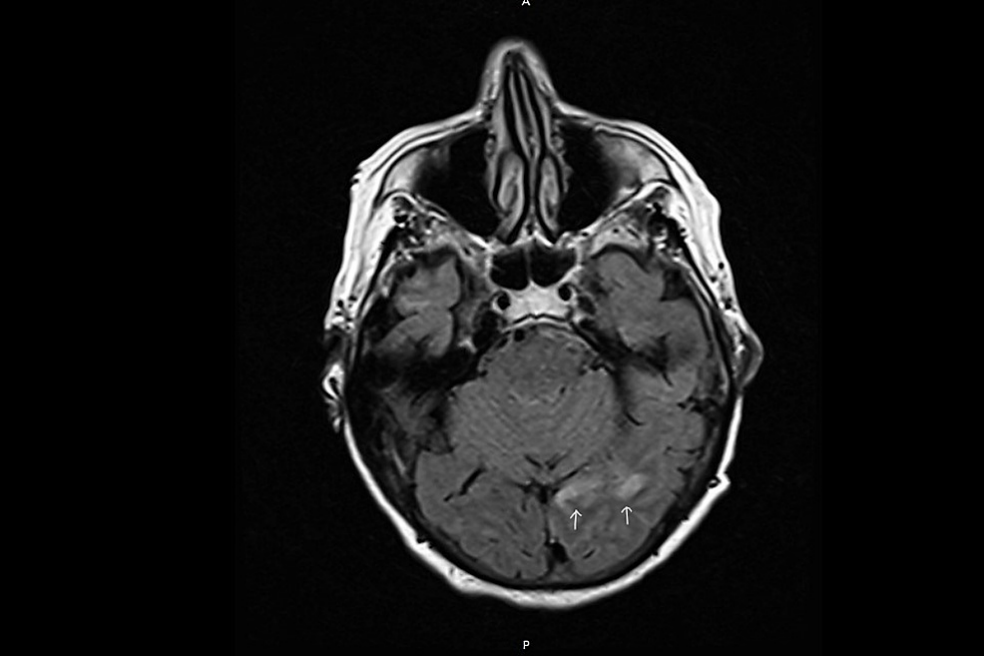
Magnetic resonance imaging of the brain without contrast. Magnetic resonance imaging (MRI) of the brain without contrast is demonstrating an area of acute/subacute stroke (white arrows) involving the medial margins of the left occipital and left posterior temporal lobe. No associated hemorrhagic transformation is demonstrated in the area of the stroke.

MRI of the brain without contrast was done upon admission and demonstrated an area of acute/subacute stroke involving the medial margin of the left occipital and left posterior temporal lobe. No associated hemorrhagic transformation was found in the field of stroke. The MRI also confirmed a large area of old cortical infarct involving the right temporal, right frontal, right parietal, and margin of the right occipital lobe, including encephalomalacia and surrounding gliosis (Figure 1).

A color testing using the “Ishihara test” revealed undifferentiated color blindness with binocular and uniocular testing of the right and left eyes. She could not recognize some colors and was frequently unable to distinguish between different grades of the same color. The following day, a subsequent computed tomography angiography (CTA) with and without contrast of the head and neck showed no major branch occlusion or focal stenosis. Transthoracic echo demonstrated a normal ejection fraction and absence of cardiac thrombus or shunts. Continuous rhythm monitoring during the hospital stay confirmed sinus rhythm. The patient reported partial improvement of her color vision throughout the two-day hospital stay. She was discharged on low-dose aspirin and high-intensity statin therapies.

## Discussion

Achromatopsia is a medical condition in which the ability to distinguish colors is impaired. Incomplete achromatopsia (dyschromatopsia) is a syndrome involving incomplete color blindness [3]. Anatomically, the brain's color center lies in the ventral occipitotemporal cortex in an area called V4 complex. However, the actual anatomical areas of the colored vision is still a matter of debate. This area is distinct from the primary visual center and has a representation of both upper and lower vision perception [4]. The most common cause of achromatopsia worldwide is congenital. Nevertheless, multiple acquired cases have been published in the medical literature [5].

Congenital achromatopsia is an autosomal recessive disorder, the prevalence of which worldwide is approximately 7% to 8%. On the other hand, acquired achromatopsia is a rare condition that tends to present subtly, often in association with visual field defects. Its recognition is challenging; thus, this medical condition's natural history is not well studied [5]. Furthermore, this condition's magnitude is not well known, given the myriad of different underlying conditions. Acquired achromatopsia cases have been reported in association with local eye diseases such as diabetic retinopathy, cataract, macular degeneration, and optic neuritis. Medications such as ethambutol and digoxin have also been linked to achromatopsia. Acquired cerebral achromatopsia was reported as a paraneoplastic syndrome where antibodies are thought to play a role in the visual cortex impairment [6]. It is also reported with multiple sclerosis cases, vitamin A deficiency, and solvent exposure [7]. Traumatic cerebral acquired achromatopsia was published for the first time in 1980 in a 24-year-old patient who presented one week after a motorcycle accident involving head trauma without wearing a protective helmet [8].

However, acquired cerebral achromatopsia in a setting of PC] strokes or other cerebral infarctions are extremely rare, and a handful of cases were reported. The first case is a 78-year-old patient who presented with temporary achromatopsia secondary to the PCA territories' bilateral ischemia [5]. The second case was reported in 1979 of a 44-year-old man with a normal color vision who suffered two cerebral infarctions of right and left occipital areas that produced severely impaired color vision [9]. PCA strokes represent around 10% of all acute ischemic strokes. Frequently, they are due to lacunar infarction but may be secondary to atherothrombotic and/or cardioembolic etiologies [10]. They can present with multiple clinical manifestations. However, visual impairment with hemianopia and macular sparing is the most common deficit. 

Our patient suffered a left occipital stroke, which clinically impacted the bilateral color vision perception. She did not have Prosopagnosia (inability to recognize the faces of familiar people), and we have not clearly excluded color agnosia. It is still unclear to us why the patient had bilateral achromatopsia despite having unilateral left PCA stroke. Our literature review found a case report in 1997, Madame D., who had two hemorrhagic strokes of the occipital areas on two different occasions and only presented with full cerebral achromatopsia after the second stroke [2]. Whether one cortex is more dominant over the other in terms of color perception is still unclear. Our patient had a stroke of the right hemisphere seven years before this presentation but never complained of achromatopsia. The patient’s bilateral achromatopsia on the presentation followed by partial improvement within two days could be explained by varying degrees of ischemia and recovery in her occipital cortex.

## Conclusions

Because PCA strokes tend to present with various non-specific symptoms, NIHSS scores tend to be lower which might delay the diagnosis and the treatment. It is frequently difficult to establish a diagnosis within the window for tissue plasminogen activator (tPA) and endovascular therapy (EVT). Additionally, Achromatopsia is a rare, subtle manifestation of PCA stroke that has not been well studied to date. Further investigation to determine the optimal treatment for and prognosis of this subset of strokes is needed. 
